# Energetics of endurance exercise in young horses determined by nuclear magnetic resonance metabolomics

**DOI:** 10.3389/fphys.2015.00198

**Published:** 2015-07-15

**Authors:** Margaux M. Luck, Laurence Le Moyec, Eric Barrey, Mohamed N. Triba, Nadia Bouchemal, Philippe Savarin, Céline Robert

**Affiliations:** ^1^Unité de Biologie Intégrative et Adaptation à l'Exercice EA 7362, Université d'Evry Val D'EssonneEvry, France; ^2^Génétique Animale et Biologie Intégrative, UMR1313, Institut National de la Recherche Agronomique (INRA)Jouy-en-Josas, France; ^3^Chimie Structures et Propriétés de Biomatériaux et d'Agents Thérapeutiques (CSPBAT), Centre National de la Recherche Scientifique, Université Paris 13, Sorbonne Paris Cité, UMR 7244Bobigny, France; ^4^Ecole Nationale Vétérinaire d'Alfort, Université Paris EstMaisons-Alfort, France

**Keywords:** horses, plasma, endurance, ^1^H NMR, metabolomics, energetics

## Abstract

Long-term endurance exercise severely affects metabolism in both human and animal athletes resulting in serious risk of metabolic disorders during or after competition. Young horses (up to 6 years old) can compete in races up to 90 km despite limited scientific knowledge of energetic metabolism responses to long distance exercise in these animals. The hypothesis of this study was that there would be a strong effect of endurance exercise on the metabolomic profiles of young horses and that the energetic metabolism response in young horses would be different from that of more experienced horses. Metabolomic profiling is a powerful method that combines Nuclear Magnetic Resonance (NMR) spectrometry with supervised Orthogonal Projection on Latent Structure (OPLS) statistical analysis. ^1^H-NMR spectra were obtained from plasma samples drawn from young horses (before and after competition). The spectra obtained before and after the race from the same horse (92 samples) were compared using OPLS. The statistical parameters showed the robustness of the model (R2Y = 0.947, Q2Y = 0.856 and cros-validated ANOVA *p* < 0.001). For confirmation of the predictive value of the model, a test set of 104 sample spectra were projected by the model, which provided perfect predictions as the area under the receiving-operator curve was 1. The metabolomic profile determined with the OPLS model showed that glycemia after the race was lower than glycemia before the race, despite the involvement of lipid and protein catabolism. An OPLS model was calculated to compare spectra obtained on plasma taken after the race from 6-year-old horses and from experienced horses (cross-validated ANOVA *p* < 0.001). The comparison of metabolomic profiles in young horses to those from experienced horses showed that experienced horses maintained their glycemia with higher levels of lactate and a decrease of plasma lipids after the race.

## Introduction

The adaptative response of energetic metabolism during endurance exercise has been extensively studied in humans and laboratory animals (Huang et al., 2010; Lewis et al., 2010; Pechlivanis et al., 2013). In human ultra-marathons, runners attain speeds of an average of 16 km.h^−1^ for runs up to 100 km and of around 6–8 km.h^−1^ in runs of 100 km or more. During endurance races, horses run at speeds ranging from 12 up to 26 km.h^−1^. At higher speeds, horses face high metabolic stress due to the intensity and duration of muscular effort. Thus, specific metabolic adaptations are needed that may be observed in horses selected for endurance for a long time, such as Arabians (Munoz et al., 2002). Intramuscular stocks of adenosine triphosphate and creatine phosphate are quickly consumed and energy must be derived from glycolytic and/or oxidative pathways (Mckenzie, 2011). Factors like diet, daily maintenance, training, or hormonal response to stress may interfere with these adaptations and consequently must be taken into account in horse management (Munoz et al., 2002, 2010). Unfortunately, despite the progress of research regarding horse exercise physiology and nutrition during endurance exercise, approximately 50% of horses that enter endurance competitions are eliminated at the veterinary control gates (vet-gates) established every 30–40 km along the ride, and about 23% of the eliminated horses exhibit serious metabolic disorders due to exhaustion during or after the ride (Nagy et al., 2010). The mechanisms involved in homeostatic disruption are dehydration, electrolytic and acid-base imbalance, substrate depletion (glycogen), hyperthermia, rhabdomyolysis, and, finally, a decreased motivation to run (Foreman, 1998). It appears that horse training, diet, and management may be improved based on a deeper understanding of equine homeostasis and energetic metabolism during long distance exercise. Improving the welfare of horses during endurance competions is essential to promoting and maintaining this pleasant equestrian discipline that is developing all around the world.

All previous epidemiological data in relation to elimination rates and their causes were obtained with experienced adult horses running in national- or international-level endurance rides (Nagy et al., 2010). There is no biological or physiological data available for 4–6-year-old horses. In humans, energetic metabolism maturation extends from childhood to adulthood (Ratel and Martin, 2012). It has been shown that aerobic metabolic capacity and recovery rate of phosphocreatine are higher in young boys than in adults. However, total power is lower, the fatigue threshold is lower, and the speed of recovery is higher for children (Ratel et al., 2006, 2008). In horses, there are no such data describing the aerobic metabolism involved in long distance exercise. As previously indicated, Arabian horses are recognized as the best breed to perform endurance competitions (Nagy et al., 2010), however their morphological development is slow and extends until the age of 6 years. Currently, there is no data available on the age-related maturation of energetic metabolism during long distance endurance training in Arabian-type horses. However, we predict that adaptations in energetic metabolism in reponse to endurance exercise are related to age in the horse as they are in humans.

The hypothesis of this study was that endurance exercise would have a strong effect on the metabolomic profiles of young horses and that adaptive responses in energetic metabolism in young horses would be different from those of more experienced horses. This metabolomic investigation in young horses will provide useful data for guiding the adaptation of ride profiles and elimination rules in relationship to age category of horses, and the improvement of horse selection, training methods, and dietary management. To test this hypothesis on age-related effects of endurance exercise on equine energetic metabolism, the primary aim of the present study was to construct a global view of the metabolome in young (6-year-old) Arabian horses competing in endurance races using a nuclear magnetic resonance (NMR) metabolomics approach. The second objective of the study was to evaluate the relationship between metabolomic profiles and performance during the race assuming that specific NMR metabolomic profiles would be identified. The final objective was to compare the metabolomic profiles obtained in 6-year-old horses after the race to the metabolomic profiles of adult experienced horses obtained previously with the same method (Le Moyec et al., 2014). By measuring changes in the levels of all small metabolites present in biological fluids, NMR metabolomics screens all metabolic pathways. As in other “Omics” methods, metabolomics allows a global investigation thanks to the combined use of NMR spectrometry and robust supervised multivariate statistical analysis (Goodpaster et al., 2010; Gebregiworgis and Powers, 2012). Briefly, comparison of metabolomic profiles before and after a 90 km endurance competition in 6-year-old horses revealed the major involvement of carbohydrate energetic pathways and high protein catabolism highlighting the potential risk for young horses to develop metabolic disorders. Comparison between young and adult experienced horses showed how the aerobic energetic pathway matures with age and accumulation of training seasons.

## Materials and methods

### Horses and measurement

The Ethics committee of the Alfort Veterinary School and the University of Paris-Est approved the study design under the number 12/07/11-1. The informed consent of the owner was obtained prior to any animal manipulation. Young horses were recruited on a voluntary basis during the national finals of the free-running speed trials for 6-year-old horses at Uzès (France) in early October of 2011, 2012, and 2013. The experienced horse population used as the reference was recruited on 160-km endurance rides and has been featured in a previous study (Le Moyec et al., 2014). All of the horses involved were Arabian or half-breed Arabian. Young horses were 6 years old while experienced horses had at least reached the age of 8.

All of the rides were divided into several sections, ranging from 20 to 40 km, depending on the total ride distance. After the completion of each section, the competitors are required to present their horses to a veterinary inspection called a “vet-gate” in which the horse has to be deemed healthy enough to be allowed to continue. In other words, any horse for which metabolic status or orthopedic conditions are judged unsatisfactory is eliminated for the animal's safety. After the horse has crossed the “vet-gate,” it is entitled to a compulsory rest period of 40–50 min before leaving for the next section of the ride.

For young horses, competitions were run in three phases of 30 km, each with a recovery period of 50 min. During these rides, their blood was sampled on two occasions for plasma NMR spectroscopy and biochemical assays. The first sample was drawn during the afternoon preceding the ride and corresponds to the before exercise (BE) sample. The second sample was drawn 30 min after the end of the ride or after disqualification during the competition and corresponds to the after exercise (AE) sample. Performance parameters of the horses were also recorded for each section of the ride.

As the samples were obtained during actual competition events, it was not possible to obtain reliable information about the diets of the competing horses. However, the usual practice is to feed the horses 2–3 h before the ride starts with a small amount of concentrate (mix of grain or pellets) and to distribute hay *ad libidum*. At each vet-gate, the horses drank water and ate about 0.5 L of concentrate and some hay, which provides electrolytes and allows the horse to keep water in the gut.

### Samples

Blood was collected with minimal restraint from a jugular vein into commercial evacuated tubes. For metabolomic analysis, blood was collected with sodium fluoride and oxalate in order to inhibit further glycolysis and the corresponding increase in lactate levels after sampling. Blood was left to decant at 4°C before plasma was separated from the pellet. Finally, plasma was stored at −80°C pending NMR analysis. For the biochemical analysis, blood was collected in dry tubes. After clotting, serum tubes were centrifuged and harvested serum was stored at 4°C until analysis, which was done within 48 h.

For plasma NMR spectroscopy, the plasma of 143 young horses was taken and a total of 196 blood samples including 107 samples before (BE) and 89 after (AE) the exercise were collected. Among them, 92 samples were collected from 46 horses finishing ride (Table 1). These last samples, taken both BE and AE from each finishing horse, were included in the young horse pair set.

**Table 1:** **Description of the young horse population indicating the number of samples in the pair set and the test set and the number of finisher horses**.

	**Young horse samples *n* = 196**			
	**Before the exercise**		**After the exercise**	
	**(BE) *n* = 107**		**(AE) *n* = 89**	
Set	Test set *n* = 61	Pair set *n* = 46	Pair set *n* = 46	Test set *n* = 43
Finishers	*n* = 13	*n* = 46	*n* = 46	*n* = 28

The 104 remaining samples were collected from different horses, either before the race (BE) for 61 of them or after the race (AE) for 43 of them. These 104 samples constituted the young horses test set. Among the BE samples of the test set, 13 horses were finishers and 48 horses were eliminated during the ride (non-finishers) and among the AE samples 28 horses were finishers and 15 were eliminated.

Concerning experienced horses, 11 finisher horses were sampled both at BE and AE for plasma NMR. This total of 22 samples was called the experienced horses pair set.

### NMR spectroscopy

The plasma samples were thawed at room temperature. In 5 mm NMR tubes, 600 μL of plasma was added to 100 μL deuterium oxide for field locking. Proton spectra were acquired at 500 MHz on a Bruker Avance III spectrometer with a 5 mm reversed QXI Z-gradient high-resolution Bruker probe. The temperature was 300 K. Free induction decays (FIDs) were acquired using a Nuclear Overhauser Effect SpectroscopY (NOESY) 1D sequence with pre-saturation (3.42.10^−5^ W) during relaxation delay (2 s) for water suppression and a 100 ms mixing time with a 90° pulse. The FIDs were collected on 32K complex points for a spectral window of 5000 Hz and 64 transients after two silent scans. The use of a NOESY1D sequence preserves lipid resonance integrity.

For young horses, a second NMR experiment was recorded for all of the samples using a Carr-Purcell-Meiboom-Gill (CPMG) 1D sequence with a tau value of 31 ms. The water signal was suppressed with a pre-saturation pulse (3.42.10^−5^ W) during relaxation delay (2 s) at the water resonance frequency. The FIDs were collected on 32K complex points for a spectral window of 5000 Hz and 64 transients after 16 silent scans. Frequently, the CPMG sequence is used to suppress the broad signal of proteins and lipids according to their short T2 relaxation times.

The FIDs were processed with NMRpipe software. Fourier transforms were performed with an exponential function producing a 0.3 Hz line broadening. Spectra were phased and a multipoint baseline correction was performed. Each spectrum was calibrated using the doublet glucose signal at 5.23 ppm. The spectral region between 0 and 9.5 ppm was divided into 9500 spectral regions of 0.001 ppm width called buckets using an in-house R software program. Each bucket was labeled with its median chemical shift value. The water region, between 4.5 and 5 ppm, was excluded. The bucket intensities were normalized using quantile normalization, as described and used previously (Kohl et al., 2012; Huang et al., 2013), in order to obtain the data X matrix for statistical analysis. Quantile normalization was used to correct the concentration effects due to possible dehydration after the ride. Unit variance scaling was performed on all variables before statistical multivariate analysis.

In the present analysis, the CPMG spectra were preferred to the NOESY 1D spectra for the investigation of the 6-year-old group samples because data interpretation was easier. The NOESY 1D spectra were kept for the comparison between the young and experienced pair set.

### Performance records

Performance parameters were assessed using the ATRM database (www.atrm-systems.fr/). Full distance and per-phase mean speed as well as heart rate at the last vet-gate and the rank of the horses were reported. Univariate statistical analysis, conducted in Excel®, between finisher and non-finisher horses were performed using Fisher's exact test (*F*-test) and Students' test (*t*-test). In finisher horses, the effect of the mean speed per phase was compared using a one-way analysis of variance test (ANOVA), followed by a Student-Newman-Keuls multiple comparison test. A *p* < 0.05 was considered to be significant.

### Biochemical assays

The sera of the young horses taken before (BE) and after (AE) the ride were assayed on an RX Imola analyzer for biochemical values of total proteins, albumin, globulin, haptoglobulin, creatinine, creatine kinase, aspartate amino transferase (ASAT), total bilirubin and serum amyloid A (SAA). The results were compared at BE vs. AE, for all horses, for finisher horses and for non-finisher horses. As the number of samples in several groups was low or the value distribution was not binomial, non-parametric statistics were used. Therefore, the results are presented with the median values and their interquartile ranges and groups are compared using the Wilcoxon test in R®. A *p* lower than 0.05 was used to reject the null hypothesis.

### Multivariate statistical analysis

PCA and OPLS analyses were performed using in-house Matlab code (Mathworks, Natick, MA) based on the Trygg and Wold method (Trygg and Wold, 2002) as previously described (Nahon et al., 2012). A principal component analysis (PCA) was first performed in the data X matrix to detect any group separation based on NMR signal variability.

A supervised multivariate statistical analysis was performed (Broadhurst and Kell, 2006; Lindon and Nicholson, 2008; Goodpaster et al., 2010; Liland, 2011). The aim of the supervised multivariate statistical analysis is to identify differences between sample spectra depending on external factors. With young horse spectra, these factors were sampling year, blood sampling time (BE/AE), performance (average speed, ranking, cardiac frequency at AE), and biochemical parameters. Each factor was encoded in a one-column matrix denoted by Y and compared through the Orthogonal projection to latent-structure analysis (OPLS) method to the data X matrix. The parameter quality for the OPLS model was assessed by the calculation of the fit parameter R2Y and cross-validated coefficient of determination Q2Y. R2Y represents the explained variance of the Y matrix. Q2Y, computed with the “leave-one-out” cross-validation method, estimates the predictability of the model. R2Y = 1 indicates perfect description of the data by the model, whereas Q2Y = 1 indicates perfect predictability. Cross-validated ANOVA (CV-ANOVA) *p*-value can be calculated as the measurement of significance for observed group separation (Eriksson et al., 2008). A *p*-value below 0.5 was considered to be a rejection of the null hypothesis i.e., groups are not separated. As another means of internal validation of the OPLS models, a permutation test (999 permutations) was performed. This evaluated whether the OPLS models, built with the samples, were significantly better than any other OPLS model obtained by randomly permuting the original sample attributes.

For the OPLS models, a score-plot and a loading plot are used to illustrate the results. Each point in the score-plot represents the projection of NMR spectrum (and thus a horse sample) on the predictive (Tpred, horizontal axis) and the first orthogonal component of the model (Torth, vertical axis). The loading plot represents the covariance between the Y-response matrix and the signal intensity of the various spectral domains. Colors were also used in the loading plot depending on the correlation between the corresponding bucket intensity and the Y variable. The metabolites were considered to be discriminating metabolites when they corresponded to the buckets with a correlation value of ≥0.5.

As proposed by Trygg and Wold (Trygg and Wold, 2002) OPLS can be used to discard unwanted variability in X. This can be done by setting the unwanted factor to Y and by removing the X variability that is predictive of Y. We used this orthogonal filtering method to remove the variability associated with the sampling year. The resulting filtered X matrix is then linearly independent of the sampling year and can be used to predict the other factors (BE/AE, performance parameters, biochemical assays). To compare the spectra of plasma sampled at BE and AE in young horses, a model was first computed with the 196 samples to determine the 90 km (or less) endurance exercise effect. In addition, in order to reduce the effects of possible confounding factors (distance ran, inter-individuals factors), another model was built with the young horse pair set (BE and AE samples taken from the same horse). The ability of this second model to discriminate between BE and AE spectra was tested using the horse spectra of the test set. This predictability was assessed by computing the area under receiver-operator characteristic curve (AUROC). The AUROC estimates the probability that the predictive component (Tpred) will rank a randomly chosen AE sample higher than a randomly chosen BE sample. A perfect discrimination corresponded to an AUROC equal to 1 (zero false positives and zero false negatives). An optimal cutoff value was also calculated which minimized both the false positive and false negative cases in the test set, assuming equal weightings on the cost of misclassification.

In order to compare the effect of exercise according to age, we suppressed inter-individual variability by using the paired method described by Westerhuis et al. (2010). In this process, paired individuals of young (young horses pair set) and experienced horses (experienced horse pair set) were used to build the original X matrix. This matrix was split into two sub-matrices: a within-X matrix and a between-X matrix. The within matrix was kept in order to eliminate the interindividual variability. Inside this within matrix, only the AE samples were used to compute an OPLS model using young vs. experienced horses as the supervising factor. There were more spectra from young horse (*n* = 46) than from experienced horse (*n* = 11) samples. To control any bias related to this unbalanced sample, we randomly selected 1000 possible combinations of 11 samples in young horses, and paired them with the 11 experienced horse samples among the initial 92 samples. This enabled the calculation of the median Q2Y calculated over all possible sample selections.

## Results

### Proton NMR spectra of plasma samples from young horses

**Figure 1:**
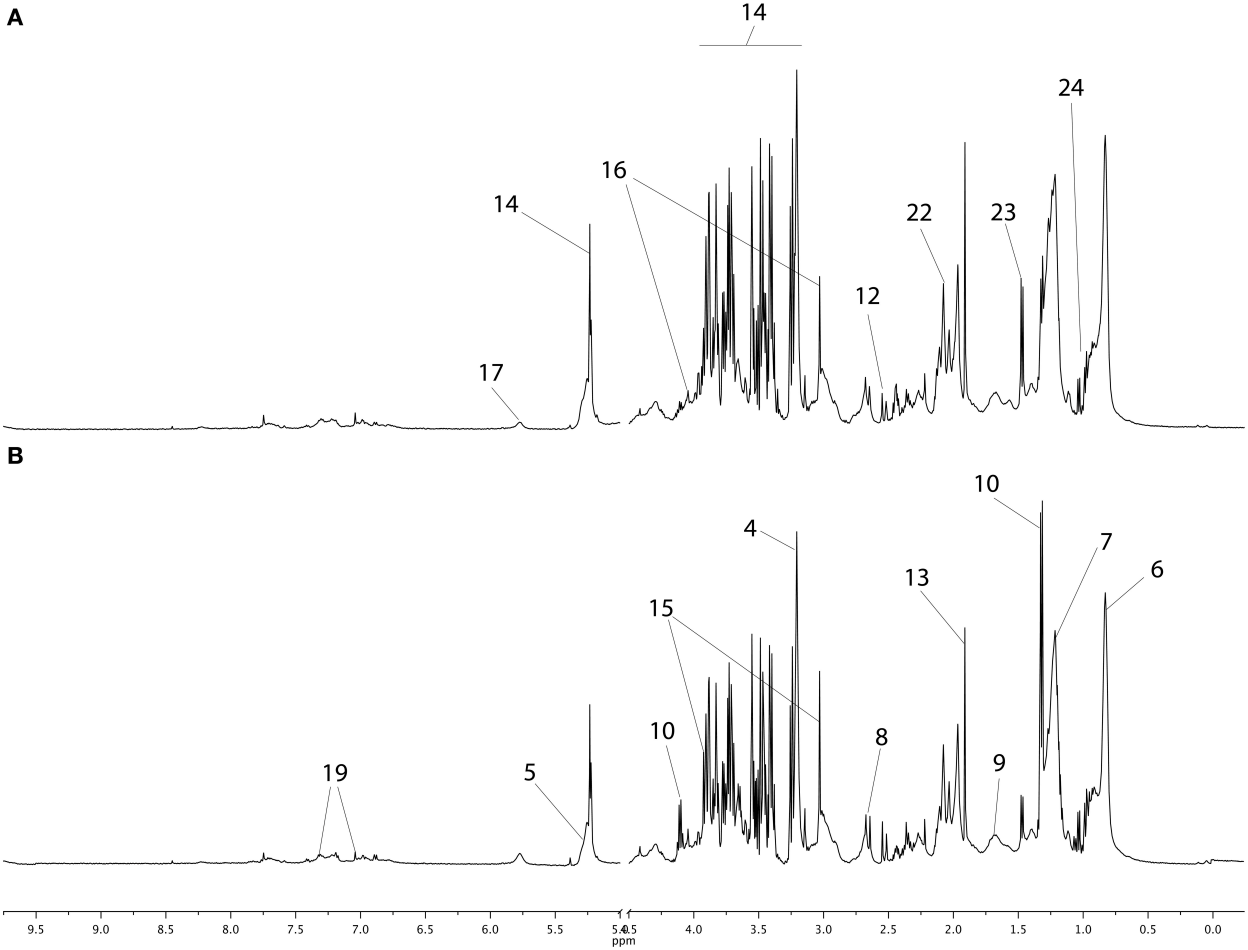
**CPMG proton 1D NMR spectra of young horse plasma samples before exercise (A) and after exercise (B)**. The main metabolites are labeled in the spectra and metabolites appearing in both spectra are not repeated. The main metabolites are labeled as follows: 1–9, metabolites from lipid metabolism; 1, β-hydroxybutyrate; 2, glycerol; 3, choline; 4, phosphocholine; 5, alkene; 6, methyl; 7, methylene; 8, methylene-α-ester; 9, methylene-β-ester; 10–14, metabolites from carbohydrate metabolism; 10, lactate; 11, fumarate; 12, citrate; 13, acetate; 14, glucose; 15–24, metabolites from amino acid metabolism and glycoproteins; 15, creatine; 16, creatinine; 17, urea; 18, phenylalanine; 19, tyrosine; 20, glutamate; 21, 2-3-methylvalerate; 22, N-Acetyl; 23, alanine; 24, branched chain amino acids (valine, leucine, isoleucine).

The NMR spectra of young horse plasma samples obtained with the CPMG sequence at BE and AE are plotted in Figure 1 with metabolite assignments. These spectra and the NOESY 1D spectra (not shown here) were similar to those reported previously (Le Moyec et al., 2014). The region between 2 and 2.1 ppm appears to be different from that for the same region in human plasma spectra. Indeed, in human plasma, only one broad resonance is detectable, while in horse plasma spectra, three peaks are detected. They have been previously assigned to N-acetyl moieties of glycoproteins and could arise from the extracellular matrix of most tissues and/or joint synovial fluid (Hodavance et al., 2007; Keller et al., 2011; Nahon et al., 2012; Le Moyec et al., 2014). Other metabolites involved in several pathways commonly found in mammalian tissues were also identified such as amino acids (alanine, tyrosine), organic acids (lactate), lipids, and carbohydrates (α- and β-glucose).

### Metabolomic profile related to the sampling year

**Figure 2:**
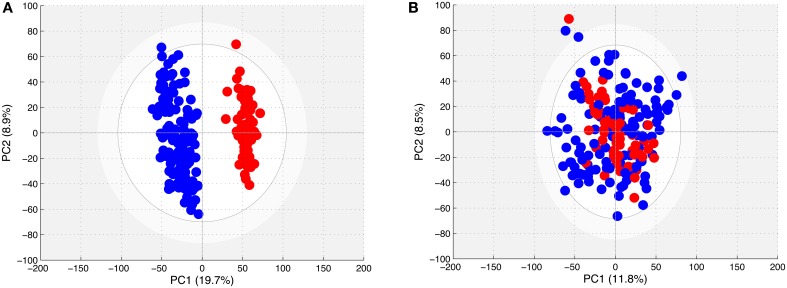
**(A)** Score plot of the PCA model computed with all spectra from samples taken from young horses. Each dot corresponds to a spectrum, colored in blue for 2011 and 2013 samples and in red for 2012 samples. **(B)** Score plot of the PCA model computed with all spectra from samples taken from young horses after removing the effect of year. Each dot corresponds to a spectrum, colored in blue for 2011 and 2013 samples and in red for 2012 samples.

The PCA models obtained with the spectra from all of the young horses (Figure 2A) showed the existence of two distinct clouds, one for the spectra of samples obtained in 2011 and 2013, and another for those obtained in 2012. However, the PCA model could not differentiate the spectra of BE horse plasma from those of AE plasma.

The OPLS models were computed with all of the sample spectra (BE and AE) in order to discriminate the plasma samples taken in 2011 and 2013 from those taken in 2012. Good statistical performances were obtained for this model as R2Y = 0.981 and Q2Y = 0.977. These fit and predictability parameters were validated with the permutation test. The loading plot (not shown here) shows that several variables were highly correlated to the sampling year (2012 year effect). It seems that the samples taken in 2012 were contaminated by a component that could be propylene glycol as the regions of propylene glycol at 3.43, 3.54, and 3.87 ppm were increased in these samples. Consequently, the data X matrix was processed in order to remove this propylene glycol component, as described previously in the materials and methods, for further analyses.

The PCA model obtained with all CPMG spectra in the filtered matrix (Figure 2B) could not differentiate the spectra according to the sampling year.

### Metabolomic profile in plasma before and after the ride

The first OPLS model was computed with the 196 samples from all horses regardless of their performance (finishers and non-finishers) in order to discriminate the metabolomic profiles before and after exercise (i.e., ≤90 km endurance exercise effect). Good statistical performances were obtained for this model as R2Y = 0.914, the Q2Y = 0.866 and the CV-ANOVA *p*-value was below 0.001. The fit (R2Y) and predictability (Q2Y) parameters were validated with the permutation test as none of the permuted models produced higher values. Several variables were highly correlated to the sampling time (BE or AE) in the loading plot of the score plot predictive axis (not shown here) of this first model. Indeed, it appeared that the general endurance exercise effects on the metabolome (Table 2) involved lipid metabolism, with an increase of signals arising from β-hydroxybutyrate, glycerol, and choline, carbohydrate metabolism, with an increase of lactate and fumarate and a decrease of glucose, and amino acid metabolism, with an increase of phenylalanine, tyrosine, glutamate, 2-hydroxy-3-methylvalerate, creatinine, and creatine.

**Table 2:** **Horse plasma metabolites discriminating the plasma profile of horses before (BE) and after (AE) the long distance endurance exercise**.

**Related metabolic pathway**	**Identified metabolites**	**Effect of exercise**	**Correlation coefficients**	
			**Model1**	**Model2**
Lipid metabolism	β-Hydroxybutyrate	↗	0.73	0.76
	Glycerol	↗	0.69	0.70
	Choline	↗	−0.54	−0.70
Carbohydrate metabolism	Lactate	↗	0.72	0.78
	Fumarate	↗	0.52	0.52
	Glucose	↘	−0.68	−0.69
Amino acid metabolism	Creatine	↗	0.58	0.53
	Creatinine	↗	0.65	0.65
	Phenylalanine	↗	0.61	0.62
	Tyrosine	↗	0.58	0.62
	Glutamate	↗	0.70	0.70
	2-hydroxy-3-methylvalerate	↗	0.87	0.89

*↗ corresponds to an increase in the AE samples when compared to the BE samples. ↘ corresponds to a decrease in AE samples when compared to BE samples. Model 1 corresponds to the ≤90 km endurance exercise effect model computed with the samples of all horses involved in the study whatever the outcome of the ride (finishers and non-finishers). Model 2 corresponds to the 90 km ride effect model computed with the young horses pair set corresponding to finisher horses taken both at BE and AE*.

**Figure 3:**
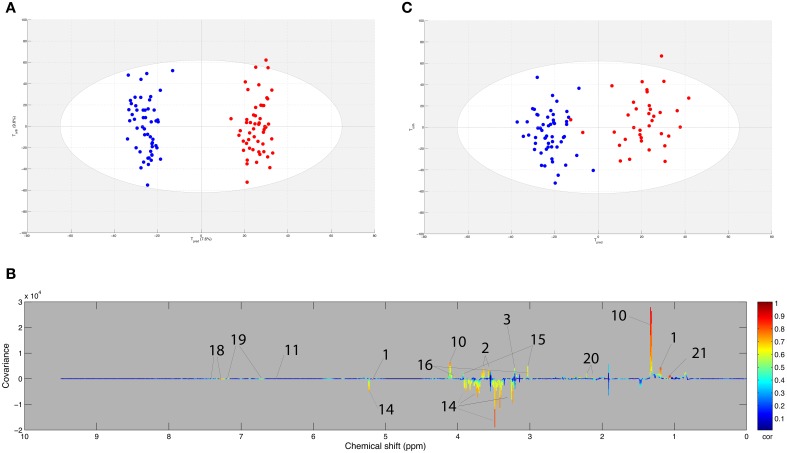
**(A)** Score plot of the OPLS model computed with before (BE) and after (AE) exercise samples from the same finisher horse. Tpred represents the predictive axis and Torth, the orthogonal axis. Each dot corresponds to a spectrum, colored in blue for BE and red for AE. **(B)** Loading plot of the score plot predictive axis. The metabolite correlations are represented by the color scale. Positive signals correspond to metabolites present at increased concentrations at AE. Conversely, negative signals correspond to metabolites present at increased concentrations at AE. The buckets are labeled according to their metabolite assignments according to Figure 1. **(C)** Projection of the sample spectra that are not in the model **(A)**. Each new spectrum was projected in the score plot using the previously constructed model **(A)** to enable prediction of BE or AE spectra. The AUROC (not shown) was equal to 1 and the optimal cutoff value was equal to 0.43.

The second OPLS model was computed with the 92 samples of the young horses pair set (i.e., finisher young horses taken both at BE and AE) in order to detect the effect of the 90 km ride. The score plot of the model is presented in Figure 3A. Good statistical performance was obtained for this model as R2Y = 0.947, Q2Y = 0.856 and the CV-ANOVA *p*-value was below 0.001. The fit and predictability parameters were validated with the permutation test. Several variables were highly correlated to the sampling time (BE or AE). These are presented in the loading plot (Figure 3B). The 90 km ride effects on the metabolome, demonstrated in the second model, were similar to the ones of the ≤90 km endurance exercise effects shown in the first model. These results are shown in Table 2.

The test set, including 104 samples, was projected into the second OPLS model. This method assessed the general predictive power of the model. As shown in Figure 3C, the model was able to assign 102 of the 104 BE and AE samples correctly to their respective groups. These two samples correspond to eliminated horses that ran only 30 km. On the whole, AUROC was equal to 1, thus indicating perfect prediction of the test set.

### Horses exercise performance and metabolomic profiles

The descriptive performance data of the samples taken at BE and AE are presented in Table 3. Unfortunately, the performance data was not available for all horses sampled for the metabolomic investigation. Briefly, for the 107 samples collected at BE, the mean speed could be assessed for 87 horses (28 non-finishers and 59 finishers) and the cardiac frequency for 84 horses (25 non-finishers and 59 finishers). For the 89 samples collected at AE, the mean speed and the cardiac frequency could be evaluated for 84 horses (10 non-finisher horses and 74 finisher horses).

**Table 3:** **Descriptive data of BE and AE samples**.

	**Total number of samples**	**Finisher horses**	**Non-finisher horses**
BE	107	59	48
Average speed (km.h^−1^)	16.4 (1.2) *n* = 87	16.4 (1.6) *n* = 59	16.3 (1.3) *n* = 28
Heart rate (bpm)	51 (4) *n* = 84	51 (4) *n* = 59	55 (6) *n* = 25
Distance (km)	77 (22)	90	58 (23)
AE	89	74	15
Average speed (km.h^−1^)	16.4 (1.0) *n* = 84	16.7 (0.8) *n* = 74	16.4 (1.0) *n* = 10
Heart rate (bpm)	52 (4) *n* = 84	52 (4) *n* = 74	52 (6) *n* = 10
Distance (km)	89 (7)	90	74 (22)

*Average speed, heart rate, and distance are given as mean values (standard deviations) calculated for the designated group of horses*.

The average speed during the ride of horses sampled at BE ranged from 12.65 to 18.92 km.h^−1^ [mean 16.35(±1.12) km.h^−1^]. In this BE set, the ranking of horses qualified after the competition ranged from the second to the 116th. The average speed during the ride of horses sampled at AE ranged from 13.90 to 18.79 km.h^−1^ [mean 16.42 (±0.99) km.h^−1^]. In this AE set, the ranking of horses qualified after the race varied from second to 116th.

These performance parameters were used as supervising factors in OPLS models. The analysis only considered spectra from samples of horses that actually finished the race. Unfortunately, OPLS models could not be computed with heart rate values at AE, rank, and mean speed. In order to improve the analysis, the mean speeds during the three phases of the ride were considered as Y supervising factors (see Table 4). The data suggest that horses were able complete the last phase significantly faster than the two previous ones. No significant differences were observed between mean speeds of phase 1 and 2. OPLS models were computed using the mean speed per phase in AE and BE samples. However, the results were inconclusive.

**Table 4:** **Performance characteristics of finisher horses sampled before (BE) and after (AE) exercise**.

	**Average speed phase 1 (km.h^−1^)**	**Average speed phase 2 (km.h^−1^)**	**Average speed phase 3 (km.h^−1^)**
BE	16.0 (1.1) *n* = 87	16.3 (1.2) *n* = 87	16.7 (2.7)^*^ *n* = 87
AE	16.1 (1.0) *n* = 84	16.3 (1.2) *n* = 84	16.9 (1.7)^*^ *n* = 84

*Data are given as mean values (standard deviations) calculated for the designated group of horses*.

* *p < 0.05 (average speed phase 3 significantly different than average speed phase 1/phase 2)*.

### Metabolomic profile and biochemical data

The median values (Interquartile Ranges) of the biochemistry assays collected at BE and AE are presented in Table 5. In AE samples, creatine kinase, creatinine, ASAT, total proteins, and haptoglobulin were all increased compared to BE samples in all horses, whether finishers or non-finishers. Globulin and haptoglobulin did not differ between BE and AE samples in any cases. Albumin differed (increased in AE samples compared to BE samples) when all horses were compared but no difference between BE and AE samples was observed when considering only the non-finisher population as opposed to the finisher population. In addition, no differences were observed when finisher and non-finisher horse samples were compared at BE and AE for all of the biochemistry assays.

**Table 5:** **Biochemical data obtained for before (BE) and after (AE) exercise samples**.

**Variables**	**Reference values**	**BE**			**AE**		
		**All horses**	**Finisher horses**	**Non-finisher horses**	**All horses**	**Finisher horses**	**Non-finisher horses**
Creatine kinase (UI/l 30°C)	150–266	160 (63.0) *n* = 106	163 (64) *n* = 59	153 (58) *n* = 47	1047^*^ (665) *n* = 84	1072^*^ (714) *n* = 71	957^*^ (357) *n* = 13
ASAT (UI/l 30°C)	195–280	246.5 (58.5) *n* = 106	247 (57) *n* = 59	246 (57.5) *n* = 47	318^*^ (72.5) *n* = 84	318^*^ (71) *n* = 71	322^*^ (68) *n* = 13
Total proteins (g/l)	50–88	69.1 (8.2) *n* = 73	69.6 (7.0) *n* = 40	68.1 (12.4) *n* = 33	75.6^*^ (9.2) *n* = 55	75.2^*^ (9.3) *n* = 46	76.3^*^ (8.0) *n* = 9
Albumin (g/l)	25–38	33.9 (2.9) *n* = 31	33.3 (2.30) *n* = 17	35.0 (2.35) *n* = 14	36.7^*^ (3.1) *n* = 30	36.2^*^ (3.10) *n* = 25	39.7 (2.80) *n* = 5
Globulin (g/l)	24–46	40.5 (6.9) *n* = 31	40.9 (5.9) *n* = 17	41.9 (7.4) *n* = 14	41 (6.4) *n* = 30	40.9 (6.4) *n* = 25	41.5 (3.6) *n* = 5
Total bilirubin (mg/l)	11.6–21.6	13.6 (5.0) *n* = 31	12.2 (2.70) *n* = 17	15.9 (10.2) *n* = 14	28.6^*^ (9.7) *n* = 84	28.5^*^ (10) *n* = 71	29.3^*^ (8.5) *n* = 13
Creatinine (mg/l)	9–20	16.1 (1.9) *n* = 31	16.4 (2.2) *n* = 17	15.3 (1.9) *n* = 14	19.2^*^ (2.6) *n* = 30	19.0^*^ (3.1) *n* = 25	20.2^*^ (1.2) *n* = 5
SAA (mg/l)	5–50	1.3 (0.1) *n* = 35	1.25 (4.0) *n* = 20	1.25 (0.04) *n* = 15	69.0^*^ (237) *n* = 82	88^*^ (243) *n* = 69	52.0^*^ (225) *n* = 13
Haptoglobulin (g/l)	0.5–2.5	1.4 (0.8) *n* = 31	1.3 (0.9) *n* = 17	1.5 (0.7) *n* = 14	1.6 (0.9) *n* = 82	1.6 (0.9) *n* = 69	1.7 (0.9) *n* = 13

Data are given as median values (Interquartile Ranges);

* *p < 0.05 (significant difference between BE and AE samples). SAA, serum amyloid A; ASAT, aspartate amino transferase. Reference values are given for information*.

Each biochemical parameter was used as a Y matrix to be correlated in an OPLS model with the NMR spectra obtained from plasma at the same moment (BE or AE). No significant model was computed with these biochemical parameters.

### Metabolomic profile in plasma between young and experienced horses

**Figure 4:**
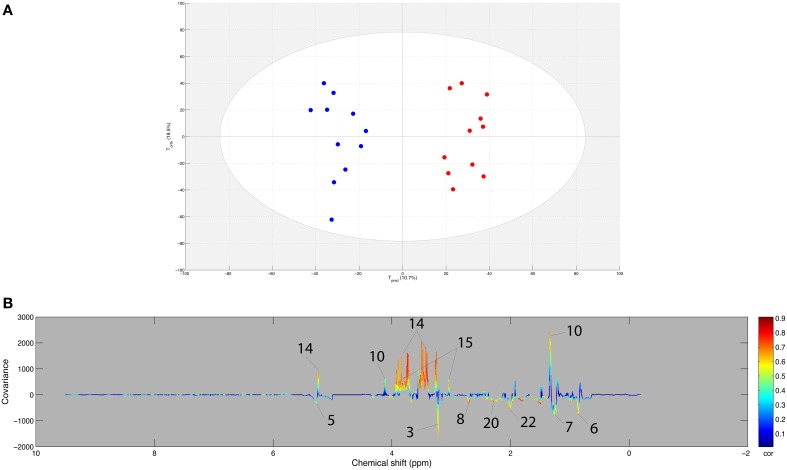
**(A)** Score plot of the best OPLS model computed with the after-exercise samples of the young and experienced horse pair set with age as supervising factor. Tpred represents the predictive axis and Torth, the orthogonal axis. Each dot corresponds to a spectrum, colored in blue for young horses and red for experienced horses. **(B)** Loading plot of the score plot predictive axis. The metabolite correlations are represented by the color scale. Positive signals correspond to an increase in the experienced horses when compared to the young horses in response to endurance exercise. Conversely, negative signals correspond to an increase in the young horses when compared to the experienced horses in response to endurance exercise. The buckets are labeled according to their metabolite assignments according to Figure 1.

An OPLS model was computed with the AE samples of the within matrix of the paired samples from both sets (young and experienced horses) using age as supervising factor. Using the resampling method, the model included the 11 samples from experienced horses and a 1000 combination of 11 samples from young horses. The score plot of the best model (Q2Y = 0.798 and R2Y = 0.896, CV-ANOVA *p* < 0.001) is presented in Figure 4A. Moreover, good statistical parameters were also obtained for the other models as the median Q2Y was 0.608. These parameters were validated with the permutation test. The loading plot of the best model (Figure 4B) shows that several variables were highly correlated to the effect of age on exercise. Indeed, it appeared that the effect of age on the response to exercise on the metabolome (Table 6) was a stronger signal arising from carbohydrate metabolism (glucose, lactate, creatine) and weaker signals arising from lipid metabolism (choline, alkene, methyl, methylene, methylene-α-ester), amino acid metabolism (glutamate), and glycoprotein metabolism (N-acetyl) for the experienced horses when compared to young horses.

**Table 6:** **Horse plasma metabolites discriminating after exercise (AE) samples of the young and experienced horses**.

**Related metabolic pathway**	**Identified metabolites**	**Experienced *vs*. 6-year-old horses**	**Correlation coefficients**
Lipid metabolism	Choline	↘	−0.60
	Alkene	↘	−0.51
	Methyl	↘	−0.61
	Methylene	↘	−0.58
	Methylene-α-ester	↘	−0.7
Carbohydrate metabolism	Lactate	↗	0.60
	Glucose	↗	0.66
Amino acid metabolism	Creatine	↗	0.59
	Glutamate	↘	−0.68
Glycoproteins	N-acetyl	↘	−0.60

*↗ corresponds to an increase in the experienced horses when compared to the 6-year-old horses in response to endurance exercise. ↘ corresponds to an increase in the 6-year-old horses when compared to the experienced horses in response to endurance exercise*.

## Discussion

The results presented in this study show that the metabolomic profiles of young horses are strongly affected by a 90 km endurance ride and that the metabolites involved are different from those observed previously for experienced horses on 160 km endurance rides. The comparison of the plasma spectra of young horses taken at BE and at AE was performed using an OPLS model. High quality statistical parameters demonstrated that the metabolome was affected during the ride. Moreover, when sample spectra not included in the model were projected, the spectra were correctly assigned to their group except for two horses which were eliminated early in the competition. The metabolic state of these horses was therefore closer to the BE profile than to the AE one.

These results were obtained with samples taken during three different events (years 2011, 2012, 2013). The statistical treatment of the year effect as proposed by Trygg and Wold (2002) seemed to be appropriate here as the sampling year effect was eliminated while preserving the effect of endurance exercise in further measurements. Using the biochemical assays as supervising factors of OPLS, no model could classify AE, and BE NMR spectra. The spectra normalization method used here was reported to minimize dilution effects (Kohl et al., 2012; Huang et al., 2013), which is not the case for biochemical results obtained with routine methods. Consequently, hydration status may have interfered with the correlation between NMR data and biochemical assays. In order to compare the endurance exercise effects on young horses to those on experienced horses, the within matrix obtained from a paired model was used and the AE samples were compared. This process removes interindividual variability, thus enabling the comparison of post-exercise effects according to age. However, considering the lower number of pairs in the experienced horse population, a resampling method was necessary to confirm the OPLS model. These results could be obtained even though the NMR spectra were acquired after a 2-year interval between measurements. This shows the reproducibility of such NMR methods. Consequently, NMR appears to be a reliable method for the comparison of samples taken with an interval of several years between them. This property is highly interesting in the diagnosis field, for example, as models may be computed and used to give the status of a new sample spectrum collected later.

The models comparing the metabolomic profile in plasma before and after the ride in 6-year-old horses showed that glycemia is lower at the end of the ride, which favors the hypothesis that a lack of carbohydrate stores is not compensated for by lipid or protein catabolism. However, lipid and protein pathways were involved, as shown by the increase of the end products of these pathways. Concerning lipid metabolism, glycerol, choline, and β-hydroxybutyrate were increased after the ride. Glycerol is produced by triglyceride degradation during lipolysis. Choline is produced from phospholipid degradation metabolism. Triglycerides and phospholipids generate energy through β-oxidation of fatty acids, releasing β-hydroxybutyrate, a ketone body. Protein metabolism is known to participate in energy supply at the end of long endurance exercises (Henriksson, 1991). This pathway is implicated in the case of these young horses as evidenced by the increase of creatine and creatinine concentrations. These metabolites eliminate the ammonia produced by the deamination of amino acids. Another amino acid catabolite is 2-hydroxy-3-methylvalerate, produced by the deamination of isoleucine, which was found in higher concentrations after the endurance event. A high level of creatine is a direct consequence of massive muscle lysis and can be considered a marker of rhabdomyolysis in blood. Moreover, the increase of serum ASAT and creatine kinase activities observed at AE confirmed the presence of rhabdomyolysis. Exercise increased the presence of amino acids such as glutamate, phenylalanine, and tyrosine as has been previously reported (van den Hoven et al., 2011). However, several other amino acids that can be detected in plasma spectra such as branched chain amino acids, alanine and lysine did not play a role in the differentiating metabolomic profile. Their release from proteins might have been compensated for by the breakdown necessary for the entry into the citric-acid cycle after deamination. Finally, lactate, a marker of anaerobic glycolysis, which is responsible for a decrease in pH that impairs muscle function and leads to fatigue, was found in higher amounts after the ride, a phenomenon already observed in experienced horses (Le Moyec et al., 2014). This lactate increase at AE could be explained by the higher speed of young horses during the last phase of ride than the two first phases or by the fact that their lactate capacities were overwhelmed. This demanding final effort involved anaerobic metabolism.

Experienced horses were better able to maintain their glycaemia than the young horses. At the same time, experienced horses used more fatty acid chains and phospholipids as fatty acid signals and choline in their plasma spectra were lower than those of young horses. With regard to protein breakdown, experienced horses had more glutamate and creatine in their plasma when compared to young horses after the ride. These differences between young and experienced horses favor the hypothesis that higher levels of lipid and protein catabolism occur in experienced horses (Li et al., 2012). However, other types of experiments are necessary in order to test the hypothesis pertaining to stable glycaemia levels observed in older horses. An investigation of enzyme activities involved in these pathways and metabolic fluxes should be completed in order to confirm that glyceamia is maintained through higher turn-over of lipids and proteins in older horses. The experienced horses showed a higher increase in lactate levels in the blood compared to young horses. Several factors may explain this difference: riders may require greater exertion from their trained horses; the total distance run may have exhausted the aerobic capacity of experienced horses; or, the lactate elimination capacity may be higher in young horses than in experienced horses. In French Trotter horses, a horse breed specialized for races conducted at trot speed, the level of blood lactate of a population sample measured during a standardized exercise test was found to be closely related to the age of the horses and their level of training (Courouce et al., 2002). During this exercise test, the velocity at which a steady state concentration of 4 mmol/L lactate (VLa4 or V4) is maintained increases with age. This suggests that the net production of blood lactate decreases in accordance to the horse's age and level of training. For human children, it has been demonstrated that their capacity to metabolize excess lactate produced during repeated exercise is greater than that of adults (Ratel et al., 2008). Finally, the levels of N-Acetyl moieties of glycoproteins were found in higher concentrations in the plasma of experienced horses after the ride compared to young horses. This is probably due to a catabolic increase of high molecular weight glycoproteins and hyaluronic acid in muscles and joints of experienced horses (Le Moyec et al., 2014). Indeed, as young horses ran over shorter distances, their muscles and joints must have been less active and consequently their catabolic products did not increase as much after the ride when compared to experienced horses.

The physiological differences between young and experienced horses, such as their “background,” training status, speed, and the distances run may influence the adaptations to endurance exercise in different ways. First, the experienced horses have completed more events and have received endurance training over a much longer period of time. Training is indeed known to improve the adaptation of skeletal muscles to endurance exercise (Serrano et al., 2000). Second, concerning speed and distance, it appeared that young horses ran shorter distances (90 km) than the experienced horses (160 km) but with a mean speed roughly 1 km.h^−1^ higher. At higher speeds, heat production is increased and there is a greater risk of exhaustion. Moreover, in finisher young horses a significant increase was found for total protein and albumin that are known to be markers of dehydration. However, the other markers of dehydration such as globulin and haptoglobulin were not in significantly higher quantities after the ride and remained stable. An inflammatory process in young horses was also observed as demonstrated by SAA and total bilirubin significantly increased at AE.

To conclude, our results showed that endurance racing consistently modifies the plasma metabolomic profiles of young horses after a long distance endurance exercise (90 km). This is true regardless of the speed during the ride and the outcome of the ride (finisher or eliminated). The metabolites differentiating young horses before the ride from young horses after the ride were different from those found previously in experienced horses. Indeed, in the case of the younger horses, a decrease in glycaemia was detected compared to the experienced horses which were able to maintain their glycaemia. The lipid and protein profiles obtained after the ride suggest that different lipid and protein metabolism occurs in the case of experienced horses; this could be a consequence of their training. However, it must be kept in mind that experienced horses ran 160 km, almost twice the distance of their younger counterparts (90 km). In this case, the glycaemia decrease observed in young horses may only be a transient state taking place before lipid metabolism compensates for the carbohydrate deficiency, followed by protein catabolism, as classically described in exercice physiology. However, while this end-point metabolomic study does suggest that such a process occurs, it was not designed to investigate these kinetic data during endurance rides. A kinetic study of the metabolism comparing young and experienced horses, using samples drawn at vet-gates, could provide answers to this question. The fact that with NMR metabolomics models could be computed with samples manipulated at different times is a very promising lead seeing as statistical models may be incremented and used as a diagnosis tools. Finally, widespread use of this technique will be required if we are to identify the biomarkers or metabolomic profiles that are the most relevant for assessment of optimal horse training. Metabolomics may also be implemented for early selection of the best young horses as well as horses' potential and fitness with respect to the correct management of high performance horses and their health and well-being.

## Conflict of interest statement

The authors declare that the research was conducted in the absence of any commercial or financial relationships that could be construed as a potential conflict of interest.
